# Social and economic consequences of the cost of obstetric and neonatal care in Lubumbashi, Democratic Republic of Congo: a mixed methods study

**DOI:** 10.1186/s12884-021-03765-x

**Published:** 2021-04-21

**Authors:** Musau Nkola Angèle, Ntambue Mukengeshayi Abel, Omewatu Mungomba Jacques, Mundongo Tshamba Henri, Malonga Kaj Françoise

**Affiliations:** 1 School of Public Health, University of Lubumbashi, Lubumbashi, Democratic Republic of Congo; 2 School of Medicine, University of Lubumbashi, Lubumbashi, Democratic Republic of Congo

**Keywords:** Consequences, Cost, Obstetric and neonatal care, Lubumbashi

## Abstract

**Background:**

The aim of this study was to explore and measure the social and economic consequences of the costs of obstetric and neonatal care in Lubumbashi, the Democratic Republic of Congo.

**Methods:**

We conducted a mixed qualitative and quantitative study in the maternity departments of health facilities in Lubumbashi. The qualitative results were based on a case study conducted in 2018 that included 14 respondents (8 mothers of newborns, 2 accompanying family members and 4 health care providers). A quantitative cross-sectional analytical study was carried out in 2019 with 411 women who gave birth at 10 referral hospitals. Data were collected for one month at each hospital, and selected mothers of newborns were included in the study only if they paid out-of-pocket and at the point of care for costs related to obstetric and neonatal care.

**Results:**

Costs for obstetric and neonatal care averaged US $77, US $207 and US $338 for simple, complicated vaginal and caesarean deliveries, respectively. These health expenditures were greater than or equal to 40% of the ability to pay for 58.4% of households. At the time of delivery, 14.1% of women giving birth did not have enough money to pay for care. Of those who did, 76.5% spent their savings. When households did not pay for care, mothers and their babies were held for a long time at the place of care. This resulted in the prolonged absence of the mother from the household, reduced household income, family conflicts, and the abandonment of the home by the spouse. At the health facility level, the increase in length of stay did not generate any additional financial benefits. Mothers no longer had confidence in nurses; they were sometimes separated from their babies, and they could not access certain prescribed medications or treatments.

**Conclusion:**

The government of the DRC should implement a mechanism for subsidizing care and associate it with a cost-sharing system. This would place the country on the path to achieving universal health coverage in improving the physical, mental and social health of mothers, their babies and their households.

**Supplementary Information:**

The online version contains supplementary material available at 10.1186/s12884-021-03765-x.

## Background

All people and communities must receive the health services they need without facing financial hardship. This is achieved through universal health coverage, one of the global objectives for sustainable development by 2030, which guarantees access to quality health services for all and protects against the financial consequences of direct payment for care. Among its strategies, the WHO recommends replacing direct payments with compulsory prepayment systems, with pooling of funds to improve access to care and protection against financial risk [1, 2].

In the Democratic Republic of Congo (DRC), direct payment for care by the users themselves is the main mode of financing the health system: more than 93% of households must pay directly out-of-pocket to gain access to health care [3]. The cost of this care is high in relation to the income of the population, and health services are therefore often beyond the reach of the poorest people [4, 5]. In fact, in the DRC, there is no financial protection mechanism for the poorest individuals, and the few health insurance plans that exist are available only to certain public or private professional companies. Prepaid systems through voluntary health insurance plans cover only 7% of the population, which is a tiny fraction of the populace [6]. This presents a major challenge for the health system in general.

For obstetric and neonatal care in particular, several studies across the country have found that the cost of providing this care is expensive for households at the time of delivery and can have catastrophic effects [7–11]. Certainly, in this study, women and their partners were the main sources of payment for maternity costs, but a significant proportion of women were assisted by nongovernmental organizations, families and churches to pay for care. This motivated the present research, as the DRC has a high fertility index of 6.6 children per woman, and 81.5% of women give birth within a hospital in the presence of qualified personnel [12, 13]. Households financing this care through direct payment are likely to face significant social and economic consequences in the short and long term, and the burden of out-of-pocket payments could be significant.

In 2015, in Lubumbashi, 16% of mothers of newborns experienced a catastrophic expenditure. In 2016, 54% of mothers of newborns were detained at the point of care for non-payment of medical costs, and this practice was accepted by the hospital as a mechanism to recover operating costs and staff salaries. Moreover, to pay for care, some women took on debt, others sold their property or lost their business income, and still others were unable to pay their rent or their children’s school fees or were forced to reduce household food consumption for two months after leaving the maternity ward. As a result of their childbirth, social relations within their households deteriorated since they were victims of poor treatment, such as verbal violence, arguments with in-laws, denial of paternity, financial deprivation, abandonment by partners, or divorce [13, 14].

In this context of the DRC, where the price of health care in general constitutes a barrier for 35% of individuals [15] and the maternal mortality ratio is still among the highest in the world [13, 14], the need for a financial protection mechanism for households is indisputable, as it would reduce financial barriers to the accessibility and use of health services and would protect individuals from social and economic consequences. However, it must necessarily be based on clear quantitative and qualitative scientific evidence, and such evidence is still insufficient [3, 16–18].

Therefore, the objective of this study was to explore and measure the social and economic consequences of the costs of obstetric and neonatal care in Lubumbashi, DRC.

## Methods

### Context

This study was carried out in Lubumbashi, the second largest city in the DRC in terms of density and socioeconomic development. It has more than 2 million inhabitants over an area of 747 km^2^. This city is the capital of the province of Haut-Katanga and includes 7 administrative communes, 11 health zones covering 9 geographic zones, and 2 special zones belonging to the military and the police. There are 594 health facilities in Lubumbashi, including 69 hospitals identified through data from the DHIS2 2018 health pyramid, and each health zone has first-line structures or health centres that provide primary care, including basic emergency obstetric and neonatal care (BEmONC), intermediate structures, and hospitals that provide complementary care, including comprehensive emergency obstetric and neonatal care (CEmONC). The city has 12 hospitals that offer referral care. Except for one of the two special zones, 10 of the 11 health zones each have at least one hospital considered a referral hospital.

These health facilities belong to a large and diverse group of institutional providers (i.e., the state, religious denominations, local NGOs, and independent parastatal and private companies). The private sector represents more than 60% of them; however, the state sector remains the majority provider in the supply of hospital care through its general reference hospitals. Maternity wards are available in nearly 2/3 of the first-line structures, in all intermediate structures and in all hospitals. Nine out of ten deliveries are attended, with a caesarean section rate of less than 2% [19, 20].

### Study design

We conducted a mixed qualitative and quantitative study (QUAL-QUAN). The study design was exploratory-sequential. Having observed in previous studies that user fees for obstetric and newborn care, although paid, were high relative to household income and could become catastrophic expenses or lead to the detention of mothers and their newborns at the point of care until their acquittal [13, 21], we hypothesized that women faced with these catastrophic expenses had to face repercussions within their households [8]. The first qualitative survey aimed to explore the daily consequences of the high cost of obstetric and neonatal care that are experienced by women who have recently given birth. The results of this study allowed us to initiate a quantitative survey to measure the burden or the consequences of the charges imposed on households.

#### Qualitative aspect

The qualitative study was carried out between May and November 2018 with 14 respondents, including 8 mothers of newborns, 2 accompanying family members and 4 health professionals providing maternity services to three health facilities, including a first-line structure and two referral hospitals. The presence of mothers detained at the point of care for non-payment of fees related to obstetric and neonatal care at the time of the survey guided the choice of referral hospitals.

All respondents were selected in a rational manner (purposive or purposeful sampling) depending on whether they agreed to participate in the study and whether they could provide relevant information. All of the selected women had to pay user fees directly at the point of care and had difficulty paying these fees. Those who were selected at the level of the reference hospitals were detained there for non-payment of these costs, and we recruited them gradually as they came to the maternity ward to give birth. The health care providers chosen were health professionals responsible for the maternity service in the health facilities concerned.

This survey was a case study. Cases were defined as any woman who experienced difficulty paying for obstetric and newborn care. The information collected from mothers was supplemented and triangulated with that provided by their accompanying family members and health care providers. The survey was carried out using semi-structured individual interviews following a pretested interview guide (Additional file 1) that was developed in French and translated into the local language (Swahili). The interviews were recorded on a Dictaphone only with the consent of the respondents and transcribed by computer.

During the interviews, the developed themes allowed the participants to express themselves freely and without constraints. In the context of this study, this refers to non-reporting and reluctance to report unpleasant facts associated with difficulties in paying for care, since without the possibility of defamation and infringement of professional secrecy, the respondents were free to express their views on relations between caregiver and patient, which, in a way, demonstrated the mediocre quality of health care and disrespectful care. Interviews were iterative for respondents, as they were contacted again until the data were saturated with interview content and validated.

From the mothers of newborns, we gathered information concerning difficulties in paying for care, changes in lifestyle within the household, relationships within the couple and the family, relationships between caregivers and the mothers of newborns and strategies for the recovery of funds.

Content analysis of all the interviews was carried out to highlight the convergences and divergences of the data. The social consequences reported are at the individual/household level and not at the community/societal level. The amounts of expenditure reported (expected or paid) by women are expressed in American dollars (US $) at the average exchange rate of 1600 Congolese francs (Fc) for 1 US dollar (US $) [average exchange rate observed in 2018].

#### Quantitative aspect

After analysing the qualitative data, we conducted an analytical quantitative cross-sectional study in the maternity departments of 10 health facilities (reference hospitals). Two referral hospitals were excluded from the study since they had fewer than 10 women who had to pay out of pocket and remit user fees at the point of care.

Data were collected in 2019, and each hospital was surveyed for a period of one month. At each health facility, the survey was exhaustive, including all women who gave birth during the study period (one month) and paid directly out-of-pocket and at the place of care for the costs of obstetric and neonatal care. These criteria allowed us to constitute a sample of 411 mothers of newborns in total.

Based on interviews structured by a pretested questionnaire (Additional file 2) and a document review of maternity cards and records, we examined the sociodemographic characteristics of the women, the type of childbirth, the complications that arose, the profile of the newborns, the spending on household consumption and health expenditures.

These health expenditures were related to the financial cost borne by women and their households for obtaining obstetric and neonatal care. These costs were calculated as the sum of direct payments related to childbirth for obstetric care (medical consultation and maternity card, the event of childbirth, surgical intervention, operating kit and dressing of operating wounds in cases of caesarean section, treatment of complications, maternity stay, purchase of prescription drugs or not) and newborn care (immediate and complete drying, temperature control, eye and cord care). Transportation used for the round trip to the maternity department and other paid costs (tips) were excluded from the health expenditure analysis because at the time of the exploratory qualitative survey, the mothers who experienced complications could not provide us with relevant information on the transport costs used. They declared that they had been taken to the hospital either in a state of unconsciousness or in a serious state of ill health to such an extent that they could not participate. Regarding tips which mothers of newborns claimed were paid to healthcare providers, we observed that health providers did not validate the information provided by the mothers of newborns. Based on the declarations of mothers of newborns, we recorded and calculated the total household expenditures that corresponded to the monthly average of the following consumption expenditures: food, clothing, schooling for children, housing, transport, visits, parties and entertainment, water, electricity, mobile phone units and other unexpected expenses. All amounts spent are expressed in US dollars (US $) at the average exchange rate of 1600 Congolese francs (Fc) for 1 US dollar (US $) [average exchange rate observed in 2019].

Quantitative data were managed and analysed with SPSS Statistics version 21.0, Epi Info Version 7 and Excel 2013 software. We used the World Health Organization (WHO) methodology described by Ke Xu to measure the burden or consequences of direct health payments [22]. Thus, for each household, this burden was calculated as the percentage of direct care payments over the household’s ability to pay. This ability to pay was equal to total expenses minus substitution expenses when these were less than or equal to food expenses. In cases where substitution expenses were greater than food expenses, the ability to pay was equal to total expenses minus food expenses.

The calculation of the burden of direct health payments enabled us, using the same methodology described by Ke Xu [22], to measure the rates of financial catastrophe (catastrophic expenditure) at thresholds of 5, 10, 20 and 40% of the households’ ability to pay, along with the impoverishment of households induced by the costs of obstetric and neonatal care. A household was considered poor (living below the poverty line) when its total expenditures were lower than its substitution expenses, and a non-poor household was impoverished when it fell below the poverty line after paying for care.

### Ethical considerations

Data were collected anonymously. To ensure anonymity, the identities of all the respondents were coded. We obtained their voluntary participation on the basis of free and informed consent. The collected and analysed data were kept confidential. The research team obtained authorizations from health authorities and health professionals who provided agreement in writing. However, free and informed consent to participate in the study was obtained verbally from mothers and their accompanying family members. The study obtained the approval of the Medical Ethics Committee of the University of Lubumbashi (CEM-UNILU: UNILU/CEM/132/2019).

## Results

### Qualitative aspect

#### Financial costs of care

The results of Table 1 were derived from the qualitative research and show the characteristics of the mothers and their newborns and the characteristics of care. We observed that the average length of stay was approximately 3 weeks and that the average cost of care was US $21.80 at the first line and US $243.10 at the hospital.

**Table 1:** Characteristics of mothers, newborns and care (qualitative survey)

Characteristics	Frequency (***n*** = 8)	Mean
Average age of mothers (in years)	8	23.6 ± 6
Average age of newborns (in weeks)	8	7 ± 6
Average length of stay (in weeks)	8	3.1 ± 3
Profession of mothers of newborns		
Unemployed	3	
Informal sector	4	
Formal sector	1	
Average monthly income per woman (US $)	8	58.6 ± 69.0
Type of birth		
complicated vaginal delivery	4	
Cesarean section	4	
Average financial cost of childbirth by health facility (US $)		
Health center (first line)		
complicated vaginal delivery	2	21.8 ± 0
General referral hospital		
complicated vaginal delivery	2	250 ± 0
Cesarean section	4	120.3 ± 113.7

#### Social costs of care and its consequences

**Fig. 1:**
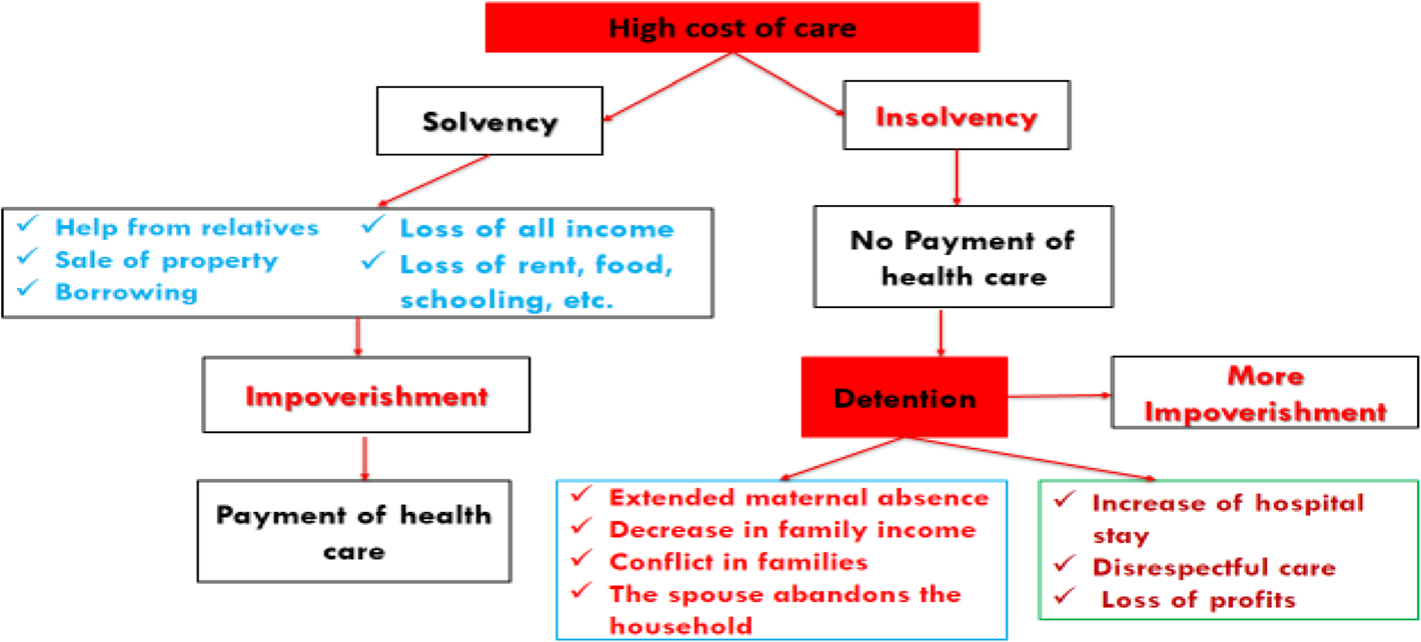
Consequences of the high cost of obstetric and newborn care on households. Graph showing what happens when the cost of obstetric and neonatal care is high (First framed with red background, black writing): Either households develop impoverishing coping strategies to pay for care (Black box, blue writings); or that mothers and their newborns are detained at the place of care because of non-payment for care (Second framed with red background, black writing). This detention is increasingly impoverishing and has consequences both for health facilities (Green box, dark red writings) and for households (Blue box, clear red writings)

Analysis of the qualitative data (Fig. 1) revealed to us that when the cost of care is too high for households, two conditions arise: if the household is solvent, it can either spend all its savings, borrow funds, sell items, reduce the consumption of essential goods (e.g., food, clothing, housing, and schooling for children) or obtain assistance from relatives. This household will be impoverished, but it will pay for care. Otherwise, if the household is insolvent and cannot obtain enough money to pay the costs, the mothers and newborns are detained for a long time at the place of care. This leads to a prolonged absence of the mother from the household, a reduction in household income, family conflicts, and the abandonment of the home by the spouse. This further impoverishes the household, and at the level of the health facility, the length of stay is sometimes increased without treatment and without the health facility recording any additional financial benefits. Patient escapes have been recorded, and the relationship between caregivers and mothers of newborns is degraded, as healthcare providers now provide disrespectful care to patients.

#### Payment difficulties and detention at the place of care

In their interviews, the health care providers and mothers of newborns said that the latter did not expect the complications they experienced during childbirth or what it would cost them. They were all hoping for a normal birth. Some women did not consult healthcare providers during pregnancy, and for others, complications were not diagnosed during prenatal visits.

*“I did not know it was a caesarean, I did not even know how much I had to pay; I was referred urgently from a health centre … My husband earns approximately 10,000 Fc (6.25 US $) per day, and we were asked to pay 350,000 Fc (219 $) for the intervention, apart from the costs relating to medication and the stay... I only gave them the 35,000 Fc ($22) that I had prepared for a normal delivery...” [Mother of newborn NIK].*

*“It is in the case of obstetric complications that the cost increases... If someone has no money available, we hold them because if we release them, there will be no hope of recovering this money …*” *[Health care providers DEPS].*

#### Changing lifestyle within the household

Mothers of newborns testified that their detention at the place of care resulted in not only separation of the couple but also prolonged maternal absence felt at the family level. This created problems for the education, nutrition, schooling, and safety of other children and reduced family income since the mother could no longer carry out her usual business activities.

*“I had to stop my small business, stay in the hospital until we pay all the bills … However, as long as I’m in the hospital, I cannot sell and I cannot help my husband: everything is messed up... He alone has to pay for everything: food, schooling, transportation, clothes, etc. Under these conditions, there will be no money to pay for care... I do not know what to do for the other children, I have seven, and they stay alone at home since their dad is a motorcyclist and he stays a long time in town to find the necessities … Only two of them go to school, my oldest daughter even got pregnant …” [Mother of newborn MAK].*

#### Changes in relationships within the couple and the family

According to the declarations of the mothers of newborns and accompanying family members, relations within the couple and the family were strained. Without knowing how long the mother would be gone, the spouse often left the family home in search of money. Family members no longer had the means to bring food, water or soap to the hospital every day. Out of shame, embarrassment or simply out of weariness, they stopped inquiring about the state of health of their sister, daughter, mother or wife and the newborn.

*“It is my aunt/my little sister who brings me food, soap or water from time to time, and most often, I share those of my room neighbours... Even my mother comes here very rarely …” [Mother of newborn VAS].*

*“Since the birth, my husband left the house on the pretext that he went to get the money... I think that sometimes he intends to return, but he cannot; he is afraid that he will be face claims to money he does not have...” [Mother of newborn CAS].*

“*… We did not argue... However, he does not come to see me at the hospital. In addition, no member of his family comes to see me; they do not even think that I should eat too … “ [Mother of newborn BEK].*

#### Changes in relationships between caregivers and mothers of newborns

In terms of health facilities, we observed that they operated at a loss, with original charges not covering the extended stay and revenue loss associated with prolonged unpaid use of bed space since the clients could no longer pay for their care. The healthcare professionals stated that beds were occupied free of charge, the drugs used were not reimbursed, the care offered was not valued, and some women who gave birth escaped. This situation led to drug shortages, demotivation of staff, lack of confidence in the patient-caregiver relationship, exclusion from certain care and disrespectful care. Indeed, because the women could not pay for care, they did not have the confidence of the nursing staff, they were detained for a long time at the place of care, they were at times separated from their babies (so that they could not escape), and they could not access certain drugs or treatments. We suggest that if the cost of care were affordable for households, this situation would probably not be observed. Nevertheless, the health professionals declared that they maintained the quality of care for mothers of newborns within the limits of their means.

*“The nurses demand money for care, no money, no care … When I donated some of the money, they also did a partial removal of the sutures …” [Mother of newborn CAS].*

*“My husband gave the pastor medical prescriptions, and he agreed to buy the medicines for us, but when we brought them to the hospital, the nurses told us that these were not the products there. I had to buy … I didn’t know what to do; I waited without treatment until my husband, who had gone in search of a better work contract, found the money for other drugs and returned to pay* …” *[Mother of newborn VAS].*

*“... They separated me from my baby and only call me to breastfeed her... I beg, I ask them to trust me, I cannot run away, I will stay in the hospital since the removal of my son’s sutures is incomplete … When the child is in neonatology without treatment, we have to pay for the bed, and the cost of care increases. I cannot escape, I just want to get my child back...” [Mother of newborn CAS].*

*“For a 10-day bill, the woman can spend 3 months in the hospital … Sometimes we traumatize them psychologically by giving them false information, but sometimes we understand that they are truly helpless and that they cannot help it....” [Health care provider DEPS].*

*“Women frequently escape without paying, especially if it is a case of stillbirth … To escape, they put their babies in a bucket or even in a bag and they run away … They simply manage with the sentries — who are not paid well either — and they go out easily... The length of stay can go up to two or three months... Sometimes, the director of the hospital authorizes discharges even in the event of partial payment of invoices … If the woman and her baby remain in the hospital beyond the planned stay, the amount of the first invoice will not change …” [Health care provider DOPK].*

*“The woman who came to give birth with a husband, she became pregnant intentionally, and she should therefore be able to pay for her care... We do not accept paupers at maternity... If necessary, the government should subsidize …” [Health care provider CEPK].*

#### Household fund recovery strategies

The respondents mentioned that they are part of poor or deprived households whose heads are unemployed, work in the informal sector (without fixed income), or practice a survival trade. No one wants to lend them money because of their insolvency.

*“I sold salt, soap and tomatoes, I earned almost 7,000 Fc to 10,000 Fc per day ($4.30 to $6.30) for the children’s food. What my husband earns helps with their education, transportation and other needs. However, since I have been here, I can no longer sell, everything is ruined... What my husband earns cannot keep the children alive at home and me too in the hospital...” [Mother of newborn NIK].*

*“There are families who obviously have the will to pay for care and to leave the hospital, but by observing them, we notice that they have nothing... It was the moms who sold tomatoes, vegetables or embers that end up in the hospital... The husband is a contractor, his salary is insignificant... If he is not taken for a daily job, he is at home and he has nothing...” [Health care provider CEPK].*

*“And the husband said to us (to the nurse): I did everything and I only found that..... Or again: I am looking for the money, but until then, here is what I’ve found … If you want you can keep the baby while you wait for the rest … We hope that people of good will, churches, politicians or other individuals come to do acts of charity and can pay for them …” [Health care provider DEPS].*

*“I sold cassava, but here I do not know how to do it anymore... My father-in-law has a bicycle that carries embers; and with his meagre means, he cannot pay all that money... “ [Mother of newborn ANN].*

### Quantitative aspect

In Table 2, the quantitative data show that the average age of the mothers of newborns recruited was 30 ± 6 years; 2 in 10 women had a primary education, and 88.8% were married. Almost half of them (46.0%) and nearly half of their spouses (45.8%) were in informal professions. Each household had an average of 3 ± 2 living children.

**Table 2:** Profile of mothers of newborns (quantitative survey)

Profile	Frequency (***n*** = 411) n (%)	Mean ± ***SD***
Age (in years)		30 ± 6
< 20	12 (2.9)	
20–35	306 (74.5)	
> 35–46	93 (22.6)	
Marital status		
In couple	365 (88.8)	
Single	46 (11.2)	
Level of study		
Primary	85 (20.7)	
Secondary	253 (61.6)	
University	73 (17.8)	
Profession		
Private enterprise	32 (7.8)	
Public company	27 (6.6)	
Liberal profession	189 (46.0)	
Unemployed	163 (39.6)	
Profession of spouses		
Private enterprise	78 (21.4)	
Public company	95 (26.0)	
Liberal profession	167 (45.8)	
Unemployed	25 (6.8)	
Place of investigation		
Kamalondo Hospital	26 (6.3)	
Kampemba Hospital	55 (13.4)	
Katuba Hospital	50 (12.2)	
Kenya Hospital	56 (13.6)	
Kisanga Hospital	32 (7.8)	
University clinics	87 (21.2)	
J Sendwe Hospital	31 (7.5)	
Mumbunda Hospital	36 (8.8)	
Ruashi Hospital	18 (4.4)	
HMR Hospital	20 (4.9)	
Number of living children		3 ± 2
None	7 (1.7)	
1 to 4	292 (71.0)	
4 to 8	104 (25.3)	
More than 8	8 (1.9)	

*SD = Standard deviation*

Table 3 shows that 4 in 10 women experienced complications during childbirth and that 3 in 10 women gave birth via caesarean section. Prolonged labour and obstructed labour were the most commonly treated complications (57.9%). The mean length of stay was higher in the case of obstetric and/or neonatal complications (up to 16 ± 23 days). One-quarter of newborns experienced complications at birth (25.5%), and 94.4% were alive at discharge from the maternity ward.

**Table 3:** Mode of Delivery, Complications, Newborn profile and Length of stay

Characteristics	Frequency (n = 411) n (%)	Stay in days mean ± SD
**Type of birth**		
Cesarean section	120 (29.2)	5 ± 24
Complicated vaginal delivery	52 (12.7)	16 ± 51
Normal delivery	239 (58.2)	14 ± 39
**Complications of childbirth**		
No	240 (58.4)	5 ± 24
Yes	172 (41.8)	15 ± 43
**Types of obstetric complications**		
Eclampsia and pre-eclampsia	21 (12.3)	8 ± 5
Hemorrhages	49 (28.7)	15 ± 53
Dystocia (extended work)	99 (57.9)	16 ± 43
Uterine ruptures	3 (1.7)	16 ± 23
**Newborns (*n*** **= 416)**		
*Sex*		
Female	177 (42.5)	
Male	239 (57.5)	
*State at birth*		
Living	388 (94.4)	
Stillborn	24 (5.8)	
Death after birth	4 (0.9)	
*Newborn complications*		
No	314 (75.5)	6 ± 17
Yes	102 (25.5)	19 ± 60

*SD = Standard deviation*

#### Health payments and monthly household expenses

**Fig. 2:**
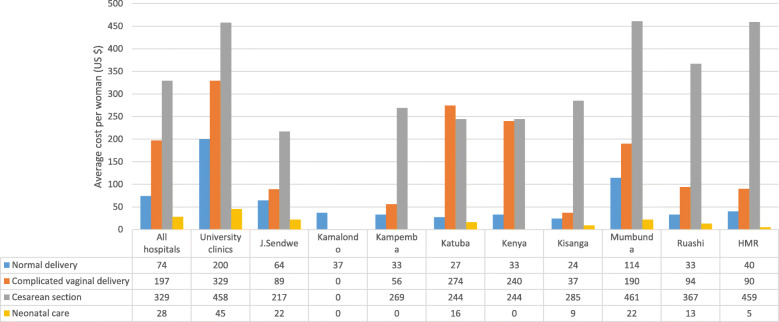
Average cost of obstetric and neonatal care per woman and per health facility (in USD $). Graph illustrating the average values of the cost of obstetric care by type of delivery, as well as the average values of the cost of neonatal care. The data is presented for all hospitals in general and for each of the 10 hospitals surveyed in particular (Data encoded, managed and analyzed with SPSS Statistics version 21.0 and Excel 2013 software)

**Fig. 3:**
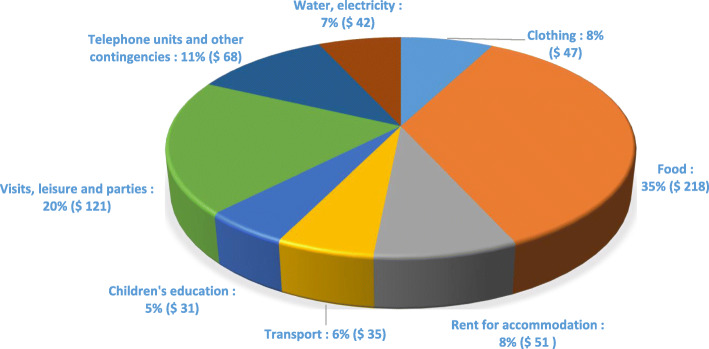
Proportion of monthly household spending (average in USD $). Graph illustrating the types of monthly household expenditure. The data are presented in the form of average values per household (USD $), as well as the proportions (%) calculated according to the total expenditure made by each household (Data encoded, managed and analyzed with SPSS Statistics version 21.0 and Excel 2013 software)

In Fig. 2, we see that the cost of care for newborns averaged US $28 per household. Moreover, the cost of obstetric care for a simple vaginal birth was 2.7 times less than that of a complicated vaginal birth (US $74 vs. US $197). The care provided during this latter type of delivery cost almost 2 times less than that in the case of a caesarean section (US $179 vs US $329). Figure 3 shows that monthly expenditures on food represented 35% of the total household expenditures, or $218 on average per household.

#### Availability of funds and adaptation strategies

At the time of delivery, 14% of women did not have enough money available to pay for care. For this reason, 9 women (2.2%) were detained at the point of care. In contrast, households that could afford care had to spend their savings in 76.5% of cases, and 17.3% of women reported decreasing consumption of essential goods; these included food expenditures in 66.2% of the cases (Table 4).

**Table 4:** Availability of funds and household adjustment mechanisms

Characteristics	Frequency = 411 n (%)
**Money available at the time of delivery**	
No	58 (14.1)
Yes	353 (85.9)
**Source of income if money available (*n*** **= 353)**	
Help from friends and relatives	16 (4.5)
Money transferred from outside by relatives	7 (1.9)
Loan	19 (5.4)
Saving	270 (76.5)
Sale of assets	41 (11.6)
**Reason for lack of funds if money not available (*n*** **= 58)**	
Spouse on a trip	4 (6.9)
Unemployed wife or spouse	8 (13.8)
Insufficient financial income	43 (74.1)
Type of care not planned	3 (5.2)
**Detention of woman for lack of money**	
Yes	9 (2.2)
No	402 (97.8)
**Reduction of basic needs to pay for care**	
No	340 (82.7)
Yes	71 (17.3)
**Types of reduced needs (if yes)**	
Children’s education	13 (18.3)
Rent	7 (9.9)
Food	47 (66.2)
Other requirements	4 (5.6)

#### Impact of health expenditures related to obstetric and newborn care on households

It was clear that almost all of the households surveyed were already poor (9 out of 10 households). Health expenditures related to obstetric and neonatal care represented 20.8, 79.6 and 121.6% of households’ ability to pay for simple deliveries, complicated vaginal deliveries and caesarean sections, respectively. However, compared with total expenditures, health expenditures represented 12.3, 43.2 and 68.2% for simple deliveries, complicated vaginal deliveries and caesarean sections, respectively (Table 5). We found that the more complications that occurred at the time of delivery, the more households suffered financial disasters. Thus, 93.3, 75 and 37.2% of households that underwent caesarean section, complicated vaginal deliveries and normal deliveries, respectively, incurred catastrophic expenses greater than or equal to 40% of their ability to pay (Table 6).

**Table 5:** Average cost (CM) of obstetric and newborn care, ability to pay and household impoverishment (in US $ per woman)

Characteristics		Cost of care per woman in US $	Total household expenditure in US $	Ability to pay per household in US $	% of average cost / Total expenditure	% of Average Cost / Ability to Pay	Proportion of poor households **	Proportion of non-poor and impoverished households
	**Frequency**	*mean ± SD*	*mean ± SD*	*mean ± SD*	*%*	*%*	*n (%)*	*n (%)*
**Type of birth**								
Normal delivery *	**239**	77 ± 70	626 ± 625	371 ± 392	12.3	20.8	226 (94.6)	0 (0)
Complicated vaginal delivery *	**52**	207 ± 128	479 ± 444	260 ± 256	43.2	79.6	49 (94.2)	0 (0)
Cesarean section *	**120**	338 ± 160	495 ± 354	278 ± 249	68.2	121.6	116 (96.7)	2 (50)

** Poverty line = substitution expenses

* Obstetric and neonatal care

**Table 6:** Incidence of catastrophic expenses by type of birth (as a % of households’ ability to pay)

All the women		Expenses ≥ 5%			Expenses ≥ 10%			Expenses ≥ 20%			Expenses ≥ 40%		
	(N)	*n*	*% ǂ*	*Mean* ± *SD (US$)*	*n*	*% ǂ*	*Mean* ± SD *(US$)*	*n*	*% ǂ*	*Mean* ± *SD (US$)*	*n*	*% ǂ*	*Mean* ± *SD (US$)*
**All treatments**	**411**	**386**	**93.9**		**351**	**85.4**		**303**	**73.7**		**240**	**58.4**	
**By type of birth**													
Normal delivery	239	214	**89.5**	83 ± 72	186	**77.8**	89 ± 74	143	**59.8**	101 ± 79	89	**37.2**	118 ± 83
Complicated vaginal delivery	52	52	**100**	207 ± 128	48	**92.3**	220 ± 125	45	**86.5**	232 ± 119	39	**75.0**	247 ± 116
Cesarean section	120	120	**100**	337 ± 160	117	**97.5**	345 ± 156	115	**95.8**	350 ± 152	112	**93.3**	354 ± 148

*ǂ: % calculated by dividing n (number of women with percentage greater than or equal to the threshold) compared to N (number of women included in the study)*

## Discussion

Direct payment for care by users compensates for shortfalls in state funding since the supply of health care generally involves financial costs to be recovered [10, 21, 23–28]. In the absence of a financial protection system, these costs become high relative to household income, reducing households’ access to care [10, 21, 23–30] and/or becoming catastrophic or impoverishing expenses [22, 31–33]. Several surveys around the world have shown that this method of payment constitutes a barrier to access to quality health care [20, 34–39] and leads to a lack of equity and universal health coverage [2, 6, 10, 14, 19, 20, 22, 23].

The same is true for interventions that reduce the case fatality rate among women with obstetric complications, which in this case cause high, unaffordable, catastrophic and impoverishing costs for households in resource-poor countries (Table 6). The literature shows that households that do not have enough money to pay for care must make a choice between becoming poorer by supporting these expenses and the consequences linked to debt repayment, stress and social tensions or give up seeking treatment and endure persistent health problems that in turn reduce the ability to work and nonetheless lead to impoverishment [24, 26, 40, 41].

In Mali, between 20.7 and 53.5% of households have incurred catastrophic expenses greater than 15 and 5% of their annual income, respectively. Marriages have dissolved due to the pressures of impoverishment and debt. Even cases of maternal death result in catastrophic expenses, and the households of escapees struggled to obtain enough food; one or more family members had to leave to look for a new job to be able to repay debts or to feed their families [41]. In Burkina Faso, households were no longer able to continue their productive activities because their capital was depleted. The women blamed themselves for having aggravated preexisting financial difficulties and engendering domestic conflicts and social tensions [3]. In Bangladesh, the wealthiest households financed care through income and savings, while the poorest households resorted to borrowing from high-interest local lenders, thereby becoming vulnerable to financial difficulties [42]. In India, the median cost of care ranged from US $11.15 for normal deliveries to US $15.90 for complicated cases [43]. Almost 15% of households spent more than 40% of their monthly income. Up to approximately one year after giving birth, women with serious maternal complications not only continued to experience significant financial repercussions but also had a higher risk of depression and more difficulty performing daily household tasks [44].

Financial catastrophes related to health care affect the poorest as well as the richest. In Ghana, 11% of households spend more than 5% of their total health care spending through out-of-pocket payments, and the poor are burdened more with catastrophic expenditures [24, 26, 40, 41]. In Nigeria, 16.4% of households experienced catastrophic health payments at a threshold of 10% of the total consumer spending, but well-off households were more likely to experience catastrophic health payments than poor households. The present study in the DRC shows that 58.4% incurred catastrophic expenses greater than or equal to 40% of their ability to pay. However, 90% of households were already poor, and 76.5% spent their savings to pay for care (Tables 4, 5 and 6).

Obstetric and neonatal care is therefore costly for Congolese households. These require catastrophic expenditures for already poor households. These financial catastrophes are not without social and economic consequences because those who can pay spend their savings or reduce their basic needs (Tables 4, 5 and 6). However, for households that cannot pay, the mothers and babies are held at the point of care, sometimes without treatment, since they cannot afford care (Fig. 1).

This study aligns with several other studies within the DRC [7, 9, 10] and elsewhere [32, 45–47], revealing that even the lowest healthcare costs are out of reach for many people (Tables 4 and 5). As a result, several social and economic consequences arise at the household level: exclusion from health care, indebtedness and high use of the informal sector, such as traditional medicines and counterfeit drugs [16, 39, 48].

It is true that the presence of qualified personnel during all deliveries is considered to be one of the most important interventions for safer births because it reduces maternal deaths and increases successful delivery of infants [41, 49–52]. As this study and several other studies have demonstrated, in poor countries, women and families who must pay out-of-pocket for the costs of care suffer physically, mentally and socially, and these distressing repercussions are linked to the unaffordable cost of the care they receive.

In an country like the DRC where seven out of ten households are poor, we believe that the Congolese State should, failing to introduce free healthcare, follow the example of several other countries [16, 30, 40, 53, 54] and create an effective and sustainable healthcare subsidy policy; favour and promote the establishment of cost-sharing and/or financial risk systems that focus on the poorest households; and improve the quality of care by involving healthcare providers but also ensure that maternity care management is based on community participation, especially that of male sexual partners, who are equally involved in conception [35, 37, 55].

The elimination of user fees may increase the number of deliveries in health facilities and the number of delivery complications treated [55, 56] and may reduce inequalities and social exclusion [57]. Obviously, special attention should be paid to the quality of care to improve health outcomes [52, 58]. Overall, when the cost of obstetric and newborn care becomes unaffordable for households, it indicates that the country is not on track to attain universal health coverage.

### Study limitations

This study has limitations. First, the small sample size of the qualitative survey affects information saturation. It also affects the calculated mean values and limits the possibility of generalizing the results of this part of the study. Second, the mothers interviewed in this study were limited to those who had experienced a complicated birth, so the perception might vary in the case of a simple vaginal birth. However, the recommendations we propose can be generalized to other similar contexts in the DRC and in other low-resource countries. Due to a lack of time and funding, we did not investigate the social and economic consequences of care at the level of health centres (first line) or at the level of private hospitals. The selection of referral hospitals belonging to the public and/or parastatal sector could have effects on the calculation of the economic costs of obstetric and neonatal care mentioned in the quantitative study. These costs may be lower or higher than the costs of care in health centres or private hospitals. Nevertheless, the selected referral hospitals offer BEmONC and CEmONC to all socio-professional categories in the DRC. We also underline the probability of a recall bias that would have affected the data collected on household income and expenditure.

## Conclusion

While providing adequate obstetric care to all women with high-risk pregnancies or complications is one of the pillars of safe motherhood, the cost of this care is too high and results in catastrophic financial outcomes that have economic consequences and social impacts on already poor households. Thus, support strategies for sharing costs and/or financial risks, providing financial protection for households and improving the quality of relationships between caregivers and patients are required.

## Supplementary Information

**Additional file 1.** Data collection guide. This is the tool used for collecting qualitative data (interview guide for mothers, family members and health professionals)

**Additional file 2.** Survey Questionnaire. This is the questionnaire used to collect quantitative data from women giving birth.

## Data Availability

All the data generated or analysed during the quantitative research are included in this published article and its additional information files. Most of the data used and/or analysed during the qualitative survey are available from the corresponding author upon reasonable request. Data will be restricted when judged by the principal investigators that privacy could be compromised.

## Abbreviations

BEmONC: Basic Emergency Obstetric and Neonatal Care
CEmONC: Comprehensive Emergency Obstetric and Neonatal Care
DRC: Democratic Republic of Congo
Fc: Congolese Francs
NGOs: Non-governmental organizations
QUAL-QUAN: Qualitative and quantitative study
SD: Standard deviation
US$: US Dollar
WHO: World Health Organization

## References

[1] Evans DB, Hsu J, Boerma T (2013). Universal health coverage and universal access. Bull World Health Organ.

[2] Organisation mondiale de la Santé. Faire des choix justes pour une couverture sanitaire universelle : rapport final du Groupe Consultatif de l’OMS sur la Couverture Sanitaire Universelle et Equitable. Organisation mondiale de la Santé. 2015;90. Available at: https://apps.who.int/iris/handle/10665/185069.

[3] Ministère de la santé publique. Plan national de développement sanitaire recadré pour la période 2019–2022 : Vers la couverture sanitaire universelle. RD Congo; 2018 p. 94. Available on: www.who.int/health_financing/universal_coverage_definition/fr

[4] Nyakude M, Ministère de la santé publique de la RD Congo. Stratégie de Renforcement du Système de Santé. 2006; 49. Available on: http://www.nyankunde.org/documentation/SRSS VERSION FINALE.pdf.

[5] Ministère de la Santé. Stratégie de financement de la santé pour la couverture sanitaire universelle en RDC. RD Congo: Ministère de la Santé; Kinshasa, 2018.

[6] Ministère de la Santé. Plan national de développement sanitaire 2011-2015. RD Congo: Ministère de la Santé; Kinshasa, 2010.

[7] Musau NA, Ntambue MA, Ilunga KS, Matungulu MC, Ilunga MT, Mundongo TH, et al. The cost of obstetric and neonatal care: case study of the Jason Sendwe hospital maternity in Lubumbashi, Democratic Republic of Congo in 2015. Rev Epidemiol Sante Publique. 2018;66(2):117–24. 10.1016/j.respe.2017.11.007. 10.1016/j.respe.2017.11.007 29371034

[8] Ntambue AM, Malonga FK, Dramaix-Wilmet M, Ilunga TM, Musau AN, Matungulu CM, et al. Commercialization of obstetric and neonatal care in the Democratic Republic of the Congo: a study of the variability in user fees in Lubumbashi, 2014. PLoS One. 2018;13(10):e0205082. 10.1371/journal.pone.0205082. 10.1371/journal.pone.0205082 PMC6179261 30304060

[9] Ntambue AM, Malonga FK, Cowgill KD, Dramaix-wilmet M, Donnen P (2015). Incidence of catastrophic expenditures linked to obstetric and neonatal care at 92 facilities in Lubumbashi, Democratic Republic of the Congo. BMC Public Health.

[10] Danielle D, O’Dempsey Tim MG, Brian F (2012). User cost of Caesarean section: case study of Bunia, Democratic Republic of Congo. Int J Health Plan Manag.

[11] Cowgill KD, Ntambue A. Post-partum detention of insolvent women and their newborns in Lubumbashi, Democratic Republic of the Congo: a cross-sectional survey. The Lancet Global Health. The Author(s). Published by Elsevier Ltd. This is an Open Access article under the CC BY license; 2017;5:S4. Available on : http://linkinghub.elsevier.com/retrieve/pii/S2214109X17301110

[12] Ministère de la santé publique. Rapport OMD 2000–2015. Evaleuation des progrès accomplis par la République Démocratique du Congo dans la réalisation des Objectifs du Millénaire pour le développement. RD Congo, Kinshasa, 2015.

[13] Ministère de la santé publique. Enquête par grappes à indicateurs multiples. Rapport MICS 6. RD Congo, 2019.

[14] Ministère de la Santé. Enquête par grappes à indicateurs multiples. MICS-2010. RD Congo: Ministère de la Santé; 2010.

[15] Ministère du plan et suivi de la mise en oeuvre de la révolution de la modernité, Ministére de la santé publique. Deuxième enquête démographique et de santé en RDC 2013-2014. 2014;113–75.

[16] Criel B. Faisabilité de la mise en oeuvre de Mutuelles de Santé en République Démocratique du Congo. Belgique: Institut de Médecine Tropical d’Anvers; 2004. Available on: http://www.ilo.org/gimi/gess/RessShowRessource.do?ressourceId=147

[17] Criel B. Etude des mutuelles de santé en RDC dans le cadre de la couverture sanitaire universelle. Belgique: Institut de Médecine Tropical d’Anvers; 2016.

[18] Ministère de la Santé. Plan national de développement sanitaire 2016–2020 : vers la couverture sanitaire universelle. RD Congo: Ministère de la Santé; 2016.

[19] Chenge M, Vennet J Van Der, Porignon D. La carte sanitaire de la ville de Lubumbashi, République Démocratique du Congo. Partie I : problématique de la couverture sanitaire en milieu urbain congolais. Global Health Promot; 2016; 1757-9759; Vol 17(3): 63–74; 375173. 10.1177/1757975910375173 20876184

[20] Chenge F (2013). De la nécessité d’adapter le modèle de district au contexte urbain : Exemple de la ville de Lubumbashi en RD Congo. Stud Health Serv Organ Policy.

[21] Kowalewski M, Mujinja P, Jahn A (2002). Can mothers afford maternal health care costs? User costs of maternity services in rural Tanzania. Afr J Reprod Health.

[22] Xu K, World Health Organization. Dept. of Health System Financing Expenditure and Resource Allocation. Distribution of health payments and catastrophic expenditures Methodology. FER/EIP discussion paper ; 2005; 11 p. Available on: http://whqlibdoc.who.int/hq/2005/EIP_HSF_DP_05.2.pdf

[23] De Brouwere V, Van Lerberghe W. Réduire les risques de maternité : Stratégies et Évidence Scientifique. Stud HSO&P ii Stud Health Serv Organ Policy. 2001;18.

[24] Borghi J, Federal D (2001). Le coût des soins de santé maternelle et les alternatives de financement. Réduire les risques liés à la maternité: Stratégies et évidence. Stud Health Serv Organ Policy.

[25] Asbu EZ, Masri MD, Kaissi A. Health status and health systems financing in the MENA region: roadmap to universal health coverage. Global health research and policy; 2017;1–13. 10.1186/s41256-017-0044-9 PMC5683471 29202093

[26] Dujardin B, Mine F, De Brouwere V (2014). Améliorer la santé maternelle : un guide pour l’action systémique.

[27] Graham WJ, Bell JS, Bullough CHW. L ’ assistance qualifiée à la naissance peut-elle réduire la mortalité maternelle dans les pays en développement ? Réduire les risques liés à la maternité: Stratégies et évidence, Studies in Health Services Organisation & Policy, 18, 2001. ITGPress. Belgieque: Studies in Health Services Organization & Policy; 2001. p. 1–30.

[28] Michielsen JJ, Meulemans H, Soors W, Ndiaye P, Devadasan N, De Herdt T (2010). Social protection in health: the need for a transformative dimension. Tropical Med Int Health.

[29] World Health Organization. Making a difference in countries: strategic approach to improving maternal and newborn survival and health. Making Pregnancy Safer. Available on: http://www.who.int/reproductivehealth/.

[30] Renaudin P, Prual A, Vangeenderhuysen C, Ould Abdelkader M, OEJD OMVM. Ensuring financial access to emergency obstetric care: three years of experience with Obstetric Risk Insurance in Nouakchott, Mauritania. Int J Gynaecol Obstet. 2007;2. 10.1016/j.ijgo.2007.07.006 17900588

[31] Kawabata K, Xu K, Carrin G (2002). Preventing impoverishment through protection against catastrophic health expenditure. Bull World Health Organ.

[32] Xu K, Evans DB, Kawabata K, Zeramdini R, Klavus J, Murray CJ (2003). Household catastrophic health expenditure: a multicounty analysis. Lancet..

[33] Xu K, Evans DB, Kadama P, Nabyonga J, Ogwal PO, Nabukhonzo P, et al. Understanding the impact of eliminating user fees: utilization and catastrophic health expenditures in Uganda. Soc Sci Med. 2006;62(4):866–76. 10.1016/j.socscimed.2005.07.004. 10.1016/j.socscimed.2005.07.004 16139936

[34] Ntambue AM, Donnen P, Dramaix-Wilmet M, Malonga FK (2012). Les facteurs de risque de la mortalité périnatale dans la ville de Lubumbashi en République démocratique du Congo. Rev Epidemiol Sante Publique.

[35] Ntambue MLA, Malonga KF, Dramaix-Wilmet M, Donnen P (2012). Determinants of maternal health services utilization in urban settings of the Democratic Republic of Congo--a case study of Lubumbashi City. BMC Pregnancy Childbirth.

[36] Ministère du plan. Document de la stratégie de croissance et de réduction de la pauvrété de seconde génération Volume I. Kinshasa, RDC, 2011.

[37] Kipp W, Kamugisha J, Jacobs P, Burnham G, Rubaale T (2001). User fees, health staff incentives, and service utilization in Kabarole District, Uganda. Bull World Health Organ.

[38] Organisation Mondiale de la Santé. Financement de la santé et couverture universelle. 2019. www.who.int/health_financing/universal_coverage_definition/fr. Accessed 30 Jan 2019.

[39] Médecins Sans Frontières. Sans argent, pas de soins ! L’impact des soins payants sur la santé. Médecins sans Frontières. 2008; 40.

[40] Richard F, Witter S, De Brouwere V. Réduire les barrières financières aux soins obstétricaux dans les pays à faibles ressources. ITGPress, éditeur. Belgique: Studies in Health Services Organization & Policy; 2008.

[41] Arsenault C. Accès aux soins obstétricaux d’urgence au Mali: Dépenses catastrophiques et conséquences au sein des ménages. 2012;94p.

[42] Hoque ME, Dasgupta SK, AMA NE. Household coping strategies for delivery and related healthcare cost: findings from rural Bangladesh. Trop Med Int Health. 2015:1368–75. 10.1111/tmi.12546 25982905

[43] Issac A, Chatterjee S, Srivastava A, Bhattacharyya S. Out of pocket expenditure to deliver at public health facilities in India: a cross sectional analysis. Reproductive Health; 2016; 13:99. Available on: http://reproductive-health-journal.biomedcentral.com/articles/10.1186/s12978-016-0221-1. 10.1186/s12978-016-0221-1 PMC4997742 27557904

[44] Sahu KSBB. Out-of-Pocket health expenditure and sources of financing for delivery, postpartum, and neonatal health in urban slums of Bhubaneswar, Odisha, India. Indian J Public Health. 61(2):67–7. 10.4103/ijph.IJPH_168_15 28721954

[45] Aregbeshola BS, Khan SM. Out-of-pocket payments, catastrophic health expenditure and poverty among households in Nigeria 2010. Int J Health Policy Manage. 2018;7:798-806. Available on: 10.15171/ijhpm.2018.19. 10.15171/ijhpm.2018.19 PMC6186489 30316228

[46] Akazili J, McIntyre D, Kanmiki EW, Gyapong J, Sankoh O, Ataguba JE, et al. Assessing the catastrophic effects of out-of-pocket healthcare payments prior to the uptake of a nationwide health insurance scheme in Ghana. Global Health Action. Taylor & Francis; 2017;10. Available on: 10.1080/16549716.2017.1289735. 10.1080/16549716.2017.1289735 PMC5496048 28485675

[47] Oudmane M, Mourji F, Ezzrari A. The impact of out-of-pocket health expenditure on household impoverishment: Evidence from Morocco 2019; 34–4. 10.1002/hpm.2848 31332829

[48] Binam JN, Nkelzok V. Prefinancement communautaire des soins de santé pour un meilleur accès des populations rurales aux services de santé de base: une estimation du consentement à prépayer des ménages au centre du Cameroun. Governing Health Systems in Africa, 2008;177–90. Available on: https://codesria.org.

[49] Réseau des Besoins non Couverts Pour les Interventions Obstétricales Majeures. L’approche des besoins non couverts pour les interventions obstétricales majeures. Etude comparative Bénin, Burkina-Faso, Haiti, Mali, Maroc, Niger, Pakistan et Tanzanie. Belgique: UON Network; 2000.

[50] Prual A (1999). Grossesse et accouchement en Afrique de l’Ouest : vers une maternité à moindre risque?. Santé Publique.

[51] Borghi J, Tagmatarchi KS, Filippi V. Les coûts des soins obstétricaux et leurs conséquences sociales et économiques pour les ménages. In: Richard F, Witter S, De Brouwere V. Réduire les barrières financières aux soins obstétricaux dans les pays à faibles ressources. Belgique: Studies in Health Services Organization & Policy, 2008;25:25–52.

[52] Mpunga Mukendi D, Chenge F, Mapatano MA, Criel B, Wembodinga G. Distribution and quality of emergency obstetric care service delivery in the Democratic Republic of the Congo: it is time to improve regulatory mechanisms. Reprod Health. 2019;16. 10.1186/s12978-019-0772-z PMC6631736 31307497

[53] Carter R, Xiong X, Kuburhanwa EC, Kimanuka F, Salumu F, Clarysse G, et al. Facility conditions, obstetric and neonatal care practices, and availability of emergency obstetric and neonatal care in 72 rural health facilities in the Democratic Republic of the Congo : A cross-sectional study version 2 ; peer review : 2 approved. 2019; 1–22. 10.12688/gatesopenres.12905.2 PMC6676177 31410393

[54] Witter S, Armar-klemesu M, Dieng T. Les systèmes nationaux d’exemption des coûts liés à l’accouchement: comparaison des expériences récentes du Ghana et du Sénégal 2008;185–221.

[55] Bhattacharyya S, Srivastava A, Roy R, Avan BI. Factors influencing women’s preference for health facility deliveries in Jharkhand state, India: A cross sectional analysis. BMC Pregnancy Childbirth; 2016;16:1–9. Available: 10.1186/s12884-016-0839-6 10.1186/s12884-016-0839-6 PMC4782569 26951787

[56] Dzakpasu S, Powell-Jackson T, Campbell OMR (2014). Impact of user fees on maternal health service utilization and related health outcomes: a systematic review. Health Policy Plan.

[57] Sochas L (2019). Women who break the rules: social exclusion and inequities in pregnancy and childbirth experiences in Zambia. Soc Sci Med.

[58] Coffey D. Costs and consequences of a cash transfer for hospital births in a rural district of Uttar Pradesh, India. Soc Sci Med. Elsevier Ltd; 2014;114:89–96. Available on: 10.1016/j.socscimed.2014.05.035 10.1016/j.socscimed.2014.05.035 PMC4122674 24911512

