# Supplementary material for: Social and economic consequences of the cost of obstetric and neonatal care in Lubumbashi, Democratic Republic of Congo: a mixed methods study
Source: BMC Pregnancy Childbirth. 2021 Apr 21;21:315. doi: 10.1186/s12884-021-03765-x (PMC8059173; doi:10.1186/s12884-021-03765-x)
Supplement: Supplementary file 1 — Additional file 1. Data collection guide. This is the tool used for collecting qualitative data (interview guide for mothers, family members and health professionals) [file 12884_2021_3765_MOESM1_ESM.docx]

**DATA COLLECTION GUIDE**

« **Social and economic consequences of the cost of obstetric and neonatal care in Lubumbashi, Democratic Republic of Congo »**

**Date of investigation : /……..../….../…………**

**Place of investigation :….……………………………..**

1. **Mother of newborn**

**Respondent's code :………………………...........**

1. When did you give birth?
2. Can you tell us about the events, the circumstances surrounding your childbirth?

- *Look for and insist on elements related to:*
  1. The type of childbirth
  2. The cost of care and the difficulties in paying for this care
  3. Detention at the point of care
  4. strategies for collecting funds to pay for care
  5. Lifestyle in the household after childbirth
  6. Relationships with partner and family after childbirth

1. For you, what is childbirth (find out what respondents think of childbirth
2. Find information on the profile of the childbirth (check the information on the maternity cards and registers)

4.1. What is your age?

4.2. What is your profession?

4.3. What is your average monthly income?

4.4. How old is your baby?

4.5. What was her birth weight?

4.6 When and how will you leave the hospital?

**Thank you very much for your participation!**

1. **Accompanying family members**

**Respondent's code :………………………...........**

1. Can you tell us about the events, the circumstances around the birth of your daughter / sister?

- *Look for and insist on elements related to (Triangulate with the information given by the mother of the newborn):*

1.1. The type of childbirth

1.2. The cost of care and the difficulties in paying for this care

1.3. Detention at the point of care

1.4. Fundraising strategies to pay for care

1.5. Lifestyle in the household after childbirth

1.6. Relationships with partner and family after childbirth

2. For you, what is childbirth? (Find out what respondents think of childbirth)

**Thank you very much for your participation!**

1. **Prestataire des soins**

**Respondent's code :………………………...........**

1. Can you tell us about the difficulties in paying for childbirth?

- *Look for and insist on elements related to:*

1.1. The cost of care and the method of payment for care (about the care you give to mothers and their babies)

2. Detention of mothers and newborns at the point of care

**Thank you very much for your participation!**
