# Supplementary material for: Social and economic consequences of the cost of obstetric and neonatal care in Lubumbashi, Democratic Republic of Congo: a mixed methods study
Source: BMC Pregnancy Childbirth. 2021 Apr 21;21:315. doi: 10.1186/s12884-021-03765-x (PMC8059173; doi:10.1186/s12884-021-03765-x)
Supplement: Supplementary file 2 — Additional file 2. Survey Questionnaire. This is the questionnaire used to collect quantitative data from women giving birth. [file 12884_2021_3765_MOESM2_ESM.docx]

| **Survey questionnaire**  “**Social and economic consequences of the cost of obstetric and neonatal care in Lubumbashi, Democratic Republic of Congo**  **Order number : /…………../**  **Date of investigation : /……../……. /…………**  **Place of birth: /……………………………...……/**  **Name of investigator : /…………………………. /** | | | | | | | | |
| --- | --- | --- | --- | --- | --- | --- | --- | --- |
|  | | | | | | | |  |
| **Mother's name initial** | **Date of Birth** | **Municipality of provenance** | **Marital status** | **level of studies** | **Number of living children** | **Occupation** | **Occupation of spouse** | **Type of birth** |
|  |  | *Lubumbashi = 1* | *Single =1* | *Primary = 1* |  | *Liberal profession =1* | *Liberal profession =1* | *Simple = 1* |
| /……………....…./ |  | *Kampemba = 2* | *Married = 2* | *Secondary =2* |  | *Private enterprise = 2* | *Private enterprise = 2* | *Complicated vaginally = 2* |
|  |  | *Kenya = 3* | *Divorcee = 3* | *University = 3* |  | *Public company = 3* | *Public company = 3* | *Caesarean = 3* |
|  |  | *Katuba = 4* | *Widow = 4* |  |  | *Unemployed = 4* | *Unemployed = 4* |  |
|  |  | *Ruashi = 5* |  |  |  |  |  |  |
| **Mother's phone number** |  | *Kamalondo = 6* |  |  |  |  |  |  |
|  |  | *Annexe = 7* |  |  |  |  |  |  |
|  |  | *Other specify* |  |  |  |  |  |  |
| /…….…………………..../ | /………/………/…….….…/ | /……………….….../ | /…….….../ | /…….….../ | /…….….../ | /…….….../ | /…….….../ | /…….….../ |

| **Delivery date** | **Expected release date** | **Complications of childbirth** | **If yes, Type of complications** | | **Amount to be paid at maternity ($)** | **Money available** | **If not why ?** | |
| --- | --- | --- | --- | --- | --- | --- | --- | --- |
|  |  | *Yes = 1* |  | |  | *Yes = 1* |  | |
|  |  | *No = 2* |  | |  | *No = 2* |  | |
| /………/………/…….…/ | /……/………/……..….…/ | /…….….../ | /…….………………………………………………………………………………..../ | | /………………………………………….………………../ | /……...….../ | /………………………………………………………………………………………………/ | |
|  |  |  |  |  |  |  |  |  |
| **If yes, source of income** | **Amount ($) for other prescriptions** | **Gender of newborn** | **Newborn State** | **Complications of the Newborn** | **If yes, Type of complications** | | **Amount payable for newborn care ($)** | **Newborn weight in grams** |
| *Saving = 1* |  | *Male = 1* | *Living = 1* | *Yes = 1* |  | |  |  |
| *Loan = 2* |  | *Female = 2* | *Stillborn = 2* | *No = 2* |  | |  |  |
| *Parents help = 3* |  |  | *Deceased = 3* |  |  | |  |  |
| *Help friends = 4* |  |  | *Incubator = 4* |  |  | |  |  |
| *Sale of assets = 5* |  |  |  |  |  | |  |  |
| *Money transferred from outside = 6* |  |  |  |  |  | |  |  |
| /…………………..….../ | /…………………………………………...………………../ | /…………....….../ | /…...………..../ | /…….….../ | /……………………………………….…………………………………………………..….../ | | /……………………..…………………../ | /…...…………………..…..……../ |

| **About how much do you spend per month in $ for Food in the household?** | **How much do you spend approximately in $ per month on clothing?** | **How much do you spend approximately in $ per month on transportation / fuel?** | **How much do you spend in $ per month on rent?** | **How much do you spend approximately in $ per year on children's education?** | **How much do you spend approximately in $ per month on Holidays, leisure and visits?** | **How much do you spend approximately in $ per month on water and electricity?** | **Other expenses you incur can go up to what amount ($)? (Specify)** | **Types of other expenses ($)** |
| --- | --- | --- | --- | --- | --- | --- | --- | --- |
|  |  |  |  |  |  |  |  |  |
| /……………………………………….……...….….../ | /……………………………………………….…….....….../ | /…………………………………........….../ | /…………………….…....….../ | /……………………………....….../ | /…………………………..…....….../ | /…………………………………....….../ | /…………………………………....….../ | /……………………………………………..…....….../ |
|  |  |  |  |  |  |  |  |  |
| **Have you reduced your consumption of food, essential goods, schooling for children to meet the costs of childbirth?** | **If yes, what have you reduced?** | | **Up to approximately how much in $ do you manage to save per month?** | | **Did the woman stay in the hospital outside of the planned stay?** | | **If the parturient stayed at the maternity hospital beyond the planned stay, give the reason** | |
| *Yes = 1*  *No = 2* |  | |  | | *Yes = 1*  *No = 2* | |  | |
| /…….….../ | /…….……………………………………………………………………………………………………………….../ | | /…….…………………………../ | | /…….….../ | | /…………………………………………………………………………………………...…/ | |

| **Information to be collected on documents (invoices, cash receipts or maternity registers)** | | | | | | | | | |  |  |  |  |
| --- | --- | --- | --- | --- | --- | --- | --- | --- | --- | --- | --- | --- | --- |
|  |  |  |  |  |  | |  |  | | | | |  |
| **Total amount paid at maternity ($)** | **Amount ($) paid for the consultation and the form** | **Amount ($) paid for the act of delivery** | | **Amount ($) paid for the transfusion** | | **Amount ($) paid for complications** | | | **Amount ($) paid for drugs** | | | **Amount ($) paid for newborn care** |  |
|  | | | | | | | | | | | | |  |
| /………………………….………..…………….……../ | /…………………..……..…………../ | /…………………………………………………………………………..…..…………../ | | /…………………………………............……..………../ | | /……………………………………..…………………..……../ | | | /………………….………………….…../ | | | /…………………………..…………...……………../ |  |
|  | | | | | | | | | | | | |  |
| **Amount ($) paid for the surgical procedure** | **Amount ($) paid for the operating kit** | **Amount ($) paid for wound dressing** | **Amount paid for the incubator** | **Amount ($) paid for the stay** | **Amount ($) paid for other items at maternity (specify)** | | | **Invoice paid on release date** | | | | **Release date** |  |
|  |  |  |  |  |  | | | *Yes = 1* | | |  | |  |
|  |  |  |  |  |  | | | *No = 2* | | |  | |  |
| /……………………………………………...….….../ | /……………………………………......….../ | /……………………………….…........….../ | /…...........................…...../ | /………….................................................….../ | /………….............................................................../ | | | | /…………..….../ | | | /……/………/………….. |  |

**Thank you very much for your participation!**
